# Optimized application of the secreted Nano-luciferase reporter system using an affinity purification strategy

**DOI:** 10.1371/journal.pone.0196617

**Published:** 2018-05-02

**Authors:** JingZhe Li, ZhiLan Guo, Takashi Sato, Bo Yuan, YanYan Ma, Dan Qian, JuYing Zhong, MengMeng Jin, Peng Huang, LuYang Che, Yi Wang, Yan Lei, ChangZhen Liu

**Affiliations:** 1 Beijing Key Laboratory of Research of Chinese Medicine on Prevention and Treatment for Major Diseases, Experimental Research Center, China Academy of Chinese Medical Sciences, Beijing, China; 2 Department of Biochemistry, School of Pharmacy, Tokyo University of Pharmacy and Life Sciences, Hachioji, Tokyo, Japan; 3 Department of Applied Biochemistry, School of Pharmacy, Tokyo University of Pharmacy & Life Sciences, Hachioji, Tokyo, Japan; 4 Department of Geriatric Endocrinology, The General Hospital of Chinese People’s Liberation Army, Beijing, China; 5 Department of Orthopaedics, The General Hospital of Chinese People’s Liberation Army, Beijing, China; International University of Health and Welfare School of Medicine, JAPAN

## Abstract

Secreted Nano-luciferase (secNluc) is a newly engineered secreted luciferase that possesses advantages of high structural stability, long half-life, and glow-type kinetics together with high light emission intensity, and thus would become one of the most valuable tools for bioluminescence assays. However, like other secreted luciferases, secNluc has to mix with the components in the conditioned medium surrounding test cells, or in the biological samples such as blood or urine after being secreted. These components may interfere with secNluc-catalyzed bioluminescence reactions and thus limit the application of the secNluc reporter system. In this study, we first examined the effects of three factors, pH, serum and residual reagents, on secNluc-catalyzed bioluminescence reactions, finding that these factors could interfere with bioluminescence reactions and result in background signal. To resolve these problems, we applied a simple affinity purification strategy in which secNluc was fused with a FLAG-tag, and anti-FLAG magnetic beads were used to catch and transfer the fusion protein to PBST, an optimal buffer for secNluc-catalyzed bioluminescence reactions that was identified in this study. The results indicated that this strategy could not only negate the interferences from serum or residual reagents and enhance the stability of light emission but also **greatly increase signal intensity through enzyme enrichment**. This strategy may contribute to biomedical studies that utilize secNluc and other secreted luciferases, especially those requiring superior sensitivity, low background noise and high reproducibility.

## Introduction

Luciferases have been widely used as convenient and powerful reporting tools in numerous biomedical researches [1,2]. These enzymes oxidize the substrates leading to emission of bioluminescence which can be observed visually using a charge-coupled device camera or measured quantitatively using a luminometer [2,3]. In the past decade, luciferase-based bioluminescence has become indispensable for monitoring of biological processes including gene expression, protein-protein interactions, cell trafficking, tumorigenesis and therapy, and high-throughput screening assays in drug discovery[4–11].

Nano-luciferase is a newly engineered luciferase enzyme that originates from the deep-sea shrimp Oplophorus gracilirostris and was structural optimized by Promega [12]. This enzyme can be used as an intracellular reporter or as a secreted reporter when it is appended with a secretion signal from human IL6 [12]. This engineered luciferase enzyme is monomeric and maintains high enzyme stability both inside living cells and in culture medium, with several days of half-life[12,13]. It utilizes coelenterazine or furimazine, a coelenterazine analog, as substrates to produce a glow-type luminescence [12]. The luminescent signal produced by Nano-luciferase is approximately 150-fold brighter than those produced by firefly or *Renilla* luciferases, which have been extensively applied in biomedical studies[13]. These advantages make Nano-luciferase an attractive tool as a reporter for bioluminescence assays. Now Nano-luciferase has already been used to generate a wide variety of transgenic organisms and cells for *in vivo* and *in vitro* studies [14–18].

Compared with intracellular luciferases, secreted luciferases are particularly advantageous in many applications, such as non-invasive quantitative assessments, real-time monitoring and high-throughput drug screening [19–24]. Therefore, secreted Nano-luciferase (secNluc) can be intensively utilized as a powerful tool in these research areas. Although secNluc has characteristics of high stability, long half-life and high signal intensity emission[12], reporter system utilizing this enzyme have several practical application limitations[25]. For example, similar to other secreted luciferases, the secretion of secNluc inevitablely leads to its mixture with some interfering components, such as serum and residual experimental reagents, that exist in conditioned medium surrounding test cells or in biological samples such as blood or urine[25,26]. Additionally, the signal intensity may not be above the limit of detection in some special circumstances, such as in experiments with low cell density. In this study, we developed a simple affinity purification strategy to separate secNluc from culture medium to negate interferences from serum or residual reagents. Meanwhile this strategy could greatly increase the signal intensity via secNluc enrichment and enhance the stability of the reaction kinetic curve by placing the luciferase in an optimal environment. This strategy may contribute to biomedical studies utilizing secNluc and other secreted luciferases, especially those requiring superior sensitivity, low background noise and high reproducibility.

## Materials and methods

### Plasmid construction

The DNA sequences encoding secNluc and secNluc-FLAG, shown in Table 1 (GenBank accession number MG009448), were synthesized by the PolePolar Biotechnology Co. LTD (Beijing, China) and cloned into the *Eco*R I and *Sal* I sites of the pDC316-EBNA vector, a generous gift from the laboratory of Dr. Bin Gao (Institute of Microbiology, CAS, China) and the *Age* I and *Eco*R I sites of the pQCXIP vector individually. The DNA sequence encoding NF-κB enhancer and TATA box (NF-κB-TATA box), shown in Table 1, was also synthesized by the PolePolar Biotechnology Co. LTD (Beijing, China), and was cloned into the *Bgl* II and *Age* I sites of the constructed pQCXIP-secNluc-FLAG vector to replace the original MCMV promoter sequence. All constructed plasmids were confirmed by DNA sequencing.

**Table 1:** Sequences of the NF-κB-TATA box, secNluc and secNluc-FLAG. (NF-κB-enhancer-TATA Box: *Bgl*IIis 1 to 6; *Age* I is 237 to 242. secNluc-FLAG: *Age* I is 1 to 6; secNluc is 7 to 610; FLAG-Taq is 611 to 652; Terminator is 653 to 655; *EcoR* I is 656 to 661.

Sequence Name	Serial No	Sequence (GenBank accession number MG009448)
**NF-κB-enhancer-TATA Box**	**1**	**AGATCTGGGA ATTTCCGGGA ATTTCCGGGA ATTTCCGGGA ATTTCCTTAA TTAAAGACTC TAGAGGGTAT ATAATGGAGC CAATGACAAG ACGCTGGGCG**
	**101**	**GGGTTTGTGT CATCATAGAA CTAAAGACAT GCAAATATAT TTCTTCCGGG GACACCGCCA GCAAACGCGA GCAACGGGCC ACGGGGATGA AGCAGCGAAG**
	**201**	**AAATCTGCAC TCGACGGTAG CGCGGGACCG GCATCCACCG GT**
**secNluc**	**1**	**ACCGGTCGCC ACCATGAACT CCTTCTCCAC AAGCGCCTTC GGTCCAGTTG CCTTCTCCCT GGGCCTGCTC CTGGTGTTGC CTGCTGCCTT CCCTGCCCCA**
**secNluc-FLAG**	**1**	**ACCGGTCGCC ACCATGAACT CCTTCTCCAC AAGCGCCTTC GGTCCAGTTG CCTTCTCCCT GGGCCTGCTC CTGGTGTTGC CTGCTGCCTT CCCTGCCCCA**
**secNluc**	**101**	**GTCTTCACAC TCGAAGATTT CGTTGGGGAC TGGCGACAGA CAGCCGGCTA CAACCTGGAC CAAGTCCTTG AACAGGGAGG TGTGTCCAGT TTGTTTCAGA**
**secNluc-FLAG**	**101**	**GTCTTCACAC TCGAAGATTT CGTTGGGGAC TGGCGACAGA CAGCCGGCTA CAACCTGGAC CAAGTCCTTG AACAGGGAGG TGTGTCCAGT TTGTTTCAGA**
**secNluc**	**201**	**ATCTCGGGGT GTCCGTAACT CCGATCCAAA GGATTGTCCT GAGCGGTGAA AATGGGCTGA AGATCGACAT CCATGTCATC ATCCCGTATG AAGGTCTGAG**
**secNluc-FLAG**	**201**	**ATCTCGGGGT GTCCGTAACT CCGATCCAAA GGATTGTCCT GAGCGGTGAA AATGGGCTGA AGATCGACAT CCATGTCATC ATCCCGTATG AAGGTCTGAG**
**secNluc**	**301**	**CGGCGACCAA ATGGGCCAGA TCGAAAAAAT TTTTAAGGTG GTGTACCCTG TGGATGATCA TCACTTTAAG GTGATCCTGC ACTATGGCAC ACTGGTAATC**
**secNluc-FLAG**	**301**	**CGGCGACCAA ATGGGCCAGA TCGAAAAAAT TTTTAAGGTG GTGTACCCTG TGGATGATCA TCACTTTAAG GTGATCCTGC ACTATGGCAC ACTGGTAATC**
**secNluc**	**401**	**GACGGGGTTA CGCCGAACAT GATCGACTAT TTCGGACGGC CGTATGAAGG CATCGCCGTG TTCGACGGCA AAAAGATCAC TGTAACAGGG ACCCTGTGGA**
**secNluc-FLAG**	**401**	**GACGGGGTTA CGCCGAACAT GATCGACTAT TTCGGACGGC CGTATGAAGG CATCGCCGTG TTCGACGGCA AAAAGATCAC TGTAACAGGG ACCCTGTGGA**
**secNluc**	**501**	**ACGGCAACAA AATTATCGAC GAGCGCCTGA TCAACCCCGA CGGCTCCCTG CTGTTCCGAG TAACCATCAA CGGAGTGACC GGCTGGCGGC TGTGCGAACG**
**secNluc-FLAG**	**501**	**ACGGCAACAA AATTATCGAC GAGCGCCTGA TCAACCCCGA CGGCTCCCTG CTGTTCCGAG TAACCATCAA CGGAGTGACC GGCTGGCGGC TGTGCGAACG**
**secNluc**	**601**	**CATTCTGGCG TAAGAATT C**
**secNluc-FLAG**	**601**	**CATTCTGGCG ACGCGTGCCG GTGATTACAA GGATGACGAT GATAAGGCCG GTTAAGAATT C**

### Cell transfection and protein preparation

HEK 293E cells were purchased from National Infrastructure of Cell Line Resource, China. GP2-293 cells were purchased from Clontech. The passage numbers of these two cells are both less than 30. HeLa, cells were purchased from the American Type Culture Collection (ATCC). HEK 293E cells were grown as suspension cultures in CD293 medium (Gibco^™^, Thermo Fisher Scientific Inc) supplemented with 1% GlutaMAX (100×) (Life Technologies). The cells were routinely maintained at exponential phase in 125-ml shaker flasks, which were agitated at 120 rpm in a humidified, 5% CO2 and water-jacketed incubator at 37°C. The PEI-mediated transfection of HEK 293E cells with the constructed pDC316-EBNA-secNluc plasmid was performed according to a previously reported method[27]. The culture medium was concentrated by ultrafiltration, dialyzed against phosphate-buffered saline (PBS, pH7.4) and then subjected to size-exclusion chromatography (Superdex 75, GE Healthcare Life Sciences). The protein was collected from the peak corresponding to approximately 13 ml and analyzed by 12% SDS-PAGE. GP2-293 and HeLa cells were cultured in a humidified incubator (5% CO2 in air) at 37°C, and maintained in DMEM containing 10% (v/v) heat-inactivated FBS. GP2-293 cells were transfected with the constructed pQCXIP-NF-κB-secNluc-FLAG plasmid using lipofectamine 2000 according to the manufacturer’s protocol (Life Technologies). Cell culture medium containing secreted luciferase was collected for further analyses. A helper plasmid, pVSV-G, was co-transfected with the constructed pQCXIP-NF-κB-secNluc-FLAG plasmid into GP2-293 cells for the preparation of retroviruses. The retroviruses were used to infect HeLa cells according to the manufacturer’s protocol (Clontech). After selection using 4μg/ml puromycin (Sigma), a monoclonal cell line stably expressing secNluc-FLAG was chosen for the TNF-α stimulation analysis experiment.

### Effects of pH, FBS and residual reagents on secNluc-catalyzed bioluminescence reactions

The DMEM and RPMI1640 pH values were adjusted to a series of values ranging from 5.0 to 9.0 in increments of 0.5. The prepared secNluc protein at a final concentration of 20 ng/ml and the substrate coelenterazine at a final concentration of 3 μmol/l were added to the modified media, and the final volume of each sample was 100 μl. Emitted bioluminescence was measured in 96-well plates using a Biotek Synergy 2 Multi-Mode Plate Reader (Biotek, USA) after the addition of substrate. To test the influence of FBS, secNluc at final concentrations ranging from 0 to 500 pg/ml and the substrate coelenterazine at a final concentration of 3 μmol/l were added to phosphate-buffered saline (PBS pH7.4) containing 0 to 10% (v/v) FBS in a total volume of 100 μl per well. Emitted bioluminescence was measured as described above. To test the influence of residual reagents, four Chinese herbal monomers, geniposide, ligustrazine, glycyrrhizic acid and puerarin were dissolved in DMSO at 5 mg/ml as 100 × stock solutions, and 1μl of each stock solution was diluted in 100 μl of PBS (pH 7.4) containing 20 ng/ml secNluc and 3 μmol/l coelenterazine for analysis. DMSO (1 μl) was used as the control. To test whether the influence existed after the secNluc was added FLAG-taq, DMEM was adjusted to designated pH value as 6.5, 7.5 and 8.5. Both of the final concentration of secNluc and secNluc-FLAG was 20 ng/ml. The concentration of substrate coelenterazine, besides all items of testing parameters and equipment was used under the uniform conditions with pH value 5.0–9.0. Glycyrrhizic acid and Puerarin were also used to evaluate the influence of FLAG-tag on the enzymatic activity of secNluc. Dissolution method and concentration of drug monomer, secNluc proteinand secNluc-FLAG protein were performed as mentioned above. Emitted bioluminescence was measured as described above.

### Optimal buffer for secNluc-catalyzed bioluminescence reactions

SecNluc at a final concentration of 5 ng/ml and the substrate coelenterazine at a final concentration of 3 μmol/l were added to 6 different buffers: DMEM, RPMI1640, DMEM containing 10% (v/v) heat-inactivated FBS (D10), RPMI1640 containing 10% (v/v) heat-inactivated FBS (R10), Phosphate-Buffered Saline (PBS), and PBS containing 0.03% (v/v) Triton X-100 (PBST). Emitted bioluminescence at the various time points was measured as described above.

### Purification of secNluc-FLAG using magnetic beads

At 24h or 48h after transfection with the constructed pQCXIP-NF-κB-secNluc-FLAG plasmid, GP2-293 cell culture medium from dishes, 24-well plates or 96-well plates were transferred to either sterilized tubes or new 96-well plates according to the reaction volume. Different amounts of M2 anti-FLAG magnetic beads (Sigma) (2.5 μl of beads for 100 μl, 2 ml or 5 ml of medium; and 10 μl of beads for 2 ml or 5 ml of medium) were added to the media, and the mixtures were incubated at room temperature for 10 minutes in a rotary mixer at 30 rpm (for tubes) or in a shaker incubator at 100 rpm (for 96-well plates). The magnetic beads were then attracted and immobilized by a magnet and the supernatants were removed by pipettes. The immobilized beads were washed with PBST and collected by magnets twice before being resuspended in 100 μl of PBST. For 96-well plates, resuspended solutions were directly subjected to the spectrometer after the addition of 3 μmol/l coelenterazine and the enzyme-catalyzed bioluminescence was measured as described above. For tubes, resuspended solutions were transferred to new 96-well plates and the bioluminescence measurement was performed as described above.

### Comparison of secNluc-catalyzed bioluminescence between systems with or without purification

Briefly, at 24 h after transfection with the constructed pQCXIP-NF-κB-secNluc-FLAG plasmid, 20 μl of GP2-293 cell cultured medium was added to 100 μl of PBST containing 3 μmol/l coelenterazine and the emitted bioluminescence was measured as described above. Simultaneously, a resuspended solution prepared according to the described purification strategy from an equal aliquot of cell culture medium was added to the same reaction buffer, and the emitted bioluminescence was measured equally. For the detection of residual reagent removal, the manipulations were similar except the addition of residual reagent group, in which different concentrations of glycyrrhizic acid were mixed with culture medium before bioluminescence measurement or magnetic bead purification.

### TNF-α stimulation analysis

A monoclonal HeLa cell line stably transfected with the NF-κB-secNluc-FLAG gene was cultured in a humidified incubator (5% CO2 in air) at 37°C and maintained in DMEM containing 10% (v/v) heat-inactivated FBS. Cells were counted and inoculated in fresh DMEM complete medium at the designated cell densities in 24-well plates. Next, 50 ng/ml of TNF-α was added to induce the activation of NF-κB signaling in the engineered cell line. After 12 hours, 10, 50 and 100 μl of culture medium from cell cultures with or without TNF-α stimulation were collected and mixed with 90, 50 and 0 μl of PBST for bioluminescence measuring. Meanwhile, another 100 μl of culture medium from cell culture with or without TNF-α stimulation was collected and subjected to magnetic bead purification, during which the beads were resuspended in 100 μl of PBST. The subsequent bioluminescence measurement was performed as described above.

## Results

### Effects of pH, FBS and residual reagents on secNluc-catalyzed bioluminescence reactions

**Fig 1:**
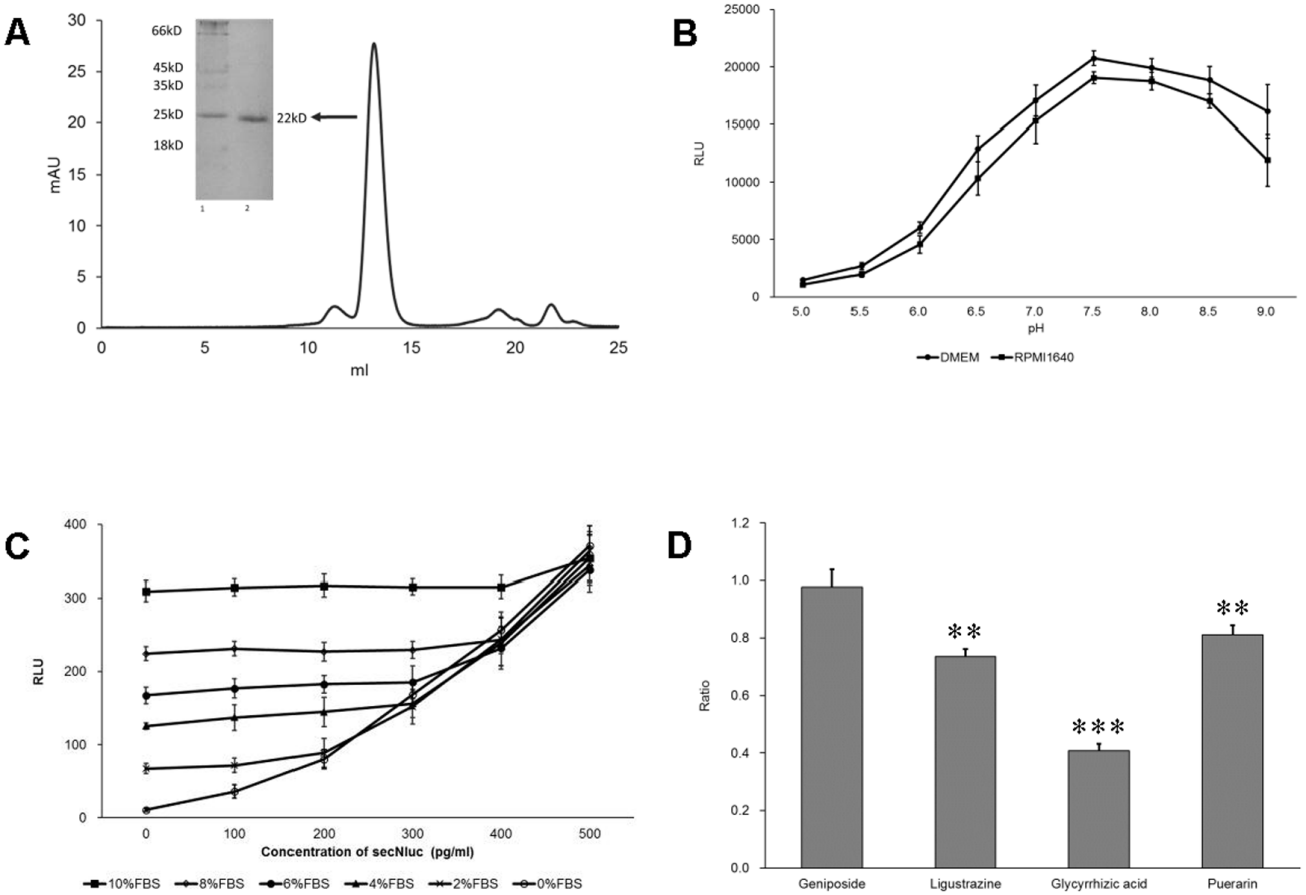
Effects of pH, FBS and residual rea gents on secNluc-catalyzed bioluminescence reactions. (A) Preparation of secNluc proteins. SecNluc proteins were secreted by HEK 293E cells transfected with the constructed pDC316-EBNA-secNluc plasmid, purified via ultrafiltration concentration and size-exclusion chromatography with the Superdex 75 column (GE Healthcare), and analyzed by 12% SDS-PAGE. Lane 1, protein marker; lane 2, purified protein. (B) Effects of DMEM and RPMI1640 media pH on secNluc-catalyzed bioluminescence reactions. The assay was performed as described in the Materials and Methods. (C) Effects of FBS on secNluc-catalyzed bioluminescence reactions. The assay was performed as described in the Materials and Methods. (D) Effects of several Chinese herbal monomers, including geniposide, ligustrazine, glycyrrhizic acid and puerarin on secNluc-catalyzed bioluminescence reactions. The reagents were used at a concentration of 50 μg/ml, and the assays were performed as described in the Materials and Methods. The ordinate values represent the ratios of RLU in the groups of Chinese herbal monomers to that in the control group. **, P< 0.01 and ***, P< 0.001. Error bars in (B) to (D) represent standard error of the mean from 3 replicate measurements.

As a newly developed secreted luciferase, secNluc is thought to own the advantages of high structural stability, long half-life, and glow-type kinetics over other secreted luciferases including the Gaussia, Metridia and Cypridina luciferases[13]. However, we wonder whether interfering factors existing in the environment would limit its application since it had to be combined with these factors after secretion. To examine this, we first prepared the purified secNluc protein. After transfecting 293E cells with the constructed pDC316-EBNA-secNluc plasmid, the secreted proteins in the culture medium were concentrated and purified using size-exclusion chromatography. SDS-PAGE analysis showed that the protein collected from the size-exclusion chromatography peak corresponding to approximately 13 ml had a molecular weight of 22 kD (Fig 1A), which is equal to the theoretical size of secNluc. The luciferase activity of the purified protein was proven in subsequent experiments.

We next examined the effects of three factors, pH, FBS and residual reagents, which are the common interfering factors existing in medium surrounding test cells, on secNluc-catalyzed bioluminescence reactions. The results showed that pH had a significant influence on bioluminescence production (Fig 1B). The light intensities exhibited comparatively little variety from pH values ranging from pH 7.0 to 8.5, with the strongest light output being observed at pH 7.5. However, when the pH of the medium was below 7.0 or over 8.5, the light intensity decreased rapidly. The result presented in Fig 1C showed that FBS could produce some luminescence signal, even in the absence of secNluc. This background signal increased as the FBS concentration increased and could shield the measured bioluminescence signals produced by secNluc-catalyzed reactions when the latter was weak. In addition, the Chinese herbal monomers, geniposide, ligustrazine, glycyrrhizic acid and puerarin, were used to mimic residual reagents. The result showed that, although geniposide had no influence on secNluc-catalyzed bioluminescence reactions, ligustrazine, glycyrrhizic acid and puerarin did have influence (Fig 1D). Specifically, 50 μg/ml glycyrrhizic acid could decrease the bioluminescence signal to approximately 40% of that of the control. These results indicated that factors in the medium, such as pH, FBS and residual reagents, might have significant effects on secNluc-catalyzed bioluminescence reactions and thus probably imit the applications of the secNluc reporter system.

### PBST was used as an optimal buffer for secNluc-catalyzed bioluminescence reactions

**Fig 2:**
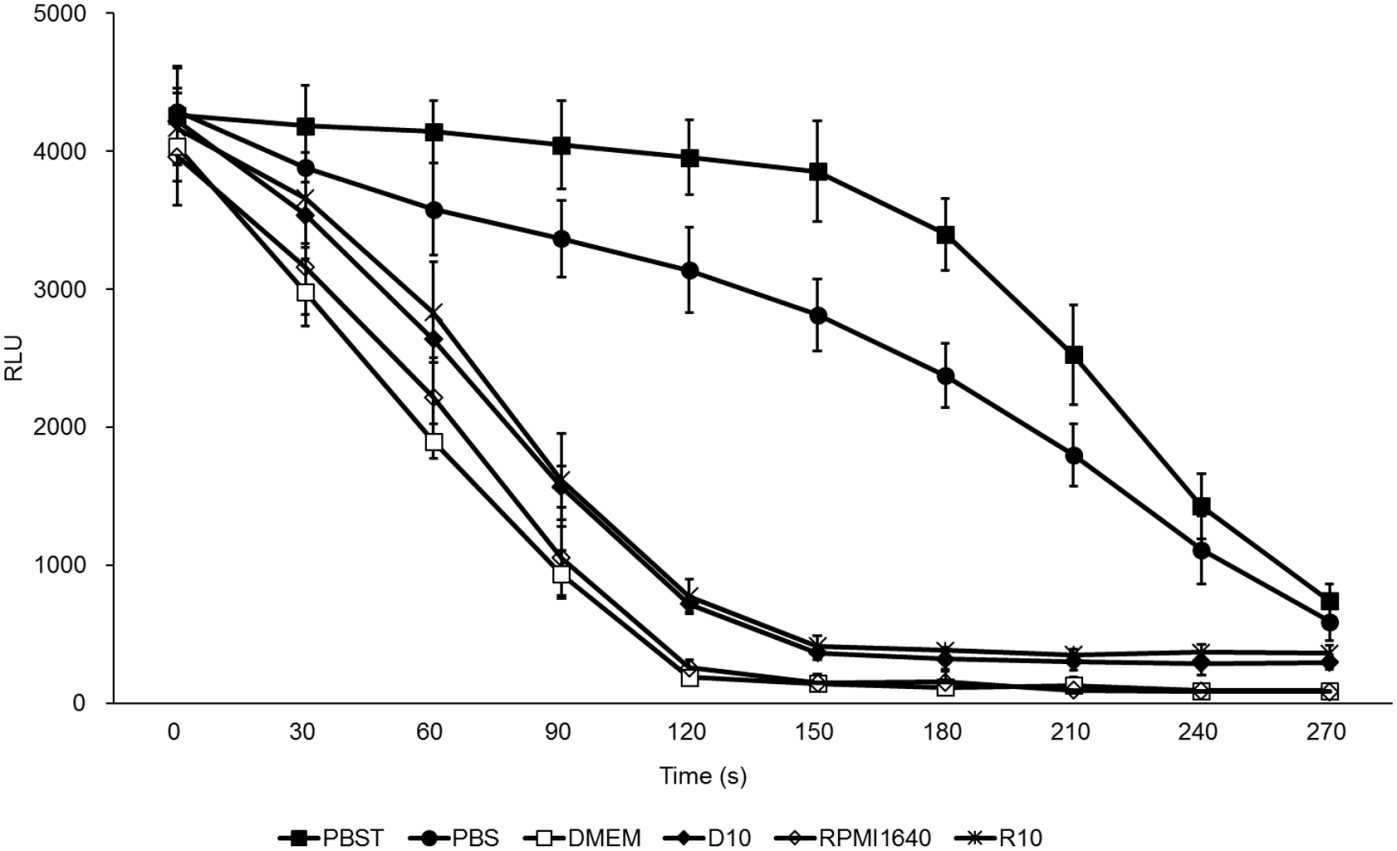
Comparison of light emission kinetics deduced from secNluc-catalyzed bioluminescence reactions in different buffers. In total, 5 ng/ml secNluc and 3 μmol/l coelenterazine were added to six buffers: DMEM, RPMI1640, D10, R10, PBS and PBST. The emitted bioluminescence at different time points (0 to 270 s at an interval of 30 s) was measured after the addition of coelenterazine in a 96-well plate by a Biotek Synergy 2 multi-mode plate reader (Biotek, USA). The data shown are the means ± standard errors of three independent experiments.

We next tried to find an optimal buffer for secNluc-catalyzed bioluminescence reactions. This optimal buffer needed to provide a stable pH and contribute to the production of stable glow-type light emission kinetics, which are important for the precision and reliability of reporter systems. Herein, we compared six buffers: DMEM, RPMI1640, D10, R10, PBS and PBST. The bioluminescence signals from the DMEM, RPMI1640, D10 and R10 groups decayed rapidly (Fig 2). The relative light unit (RLU) values at the 60s time point dropped to half of their initial values in the DMEM and RPMI1640 groups and to 65% of their initial values in the D10 and R10 groups, and the signals almost fully faded after 120 s in the DMEM and RPMI1640 groups and after 150 s in the D10 and R10 groups. In the PBS and PBST groups, however, the decay rates of the bioluminescence signals were obviously slower than those of the other four groups (Fig 2). Of the two buffers, PBST provided the best performance, with less than a 10% decrease in the RLU value being observed at 150 s, and this buffer generally met the requirements of providing a stable pH and contributing to the production of stable glow-type light emission kinetics. Therefore, we selected PBST as the optimal buffer for secNluc-catalyzed bioluminescence reactions.

### A simple affinity purification strategy for optimizing secNluc reporter system applications

**Fig 3:**
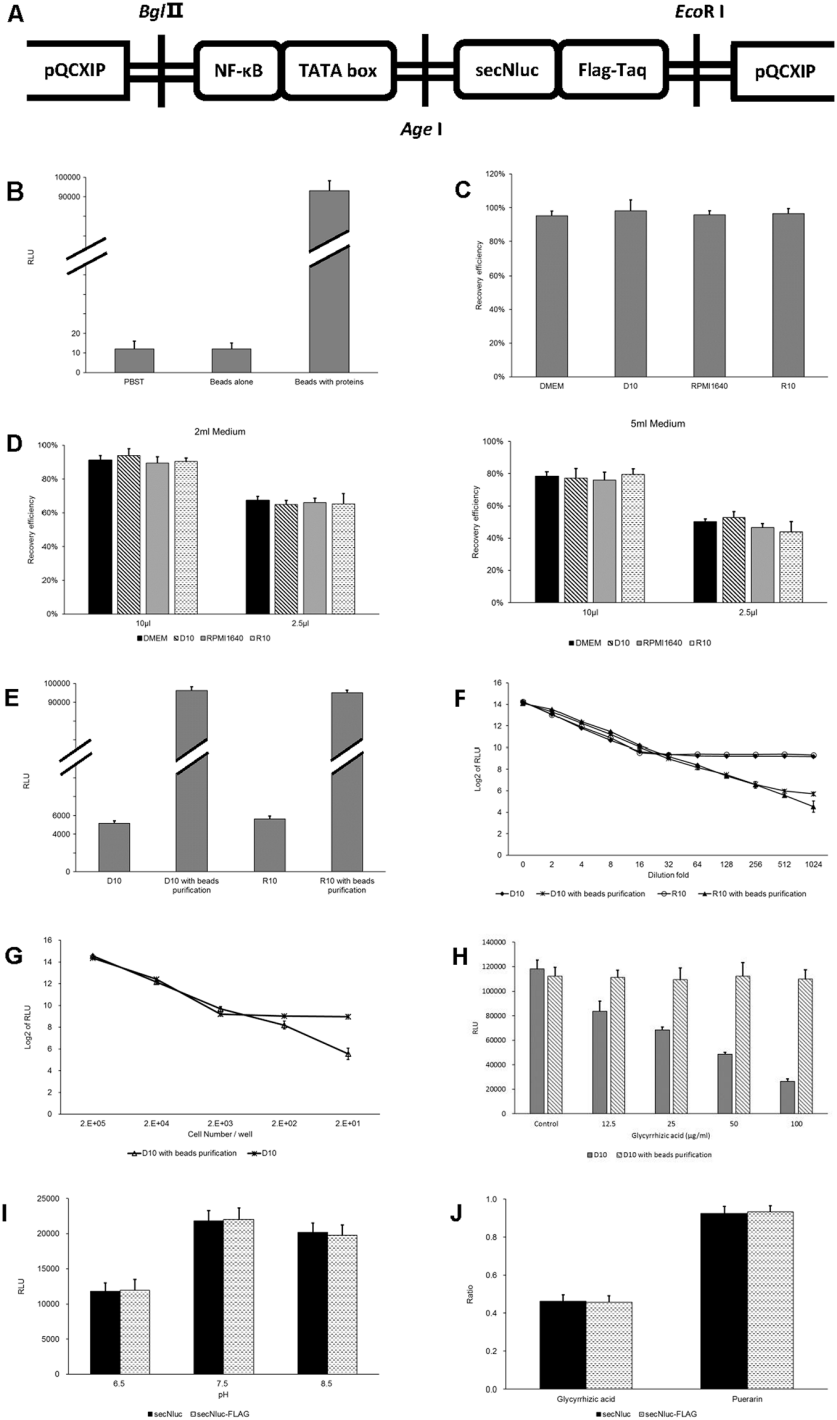
A purification strategy employing secNluc-FLAG fusion proteins and anti-FLAG magnetic beads for optimizing the application of secNluc reporter system. (A) Schematic representation of the constructed pQCXIP-NF-κB-secNluc-FLAG plasmid. (B) Luminescence signals induced by anti-FLAG beads. 2.5 μl of anti-FLAG beads were added to 100 μl of PBST containing 3 μmol/l coelenterazine to measure the luminescence signals induced by the beads. Additionally, 2.5 μl of beads binding secNluc-FLAG fusion proteins was used as the positive control, and PBST containing coelenterazine alone was used as the negative control. (C) and (D) Recovery of secNluc-FLAG proteins in different media after purification with anti-FLAG beads. 2.5 μl of beads were mixed with 100 μl of four different media containing the secreted secNluc-FLAG protein (C). Similarly, 2.5 μl and 10 μl of beads were mixed with 2 ml and 5 ml of the four different media containing the secreted secNluc-FLAG protein (D). Bead isolation and bioluminescence measurements were performed as described in the Materials and Methods. (E) Concentration of the secNluc-FLAG protein using the purification strategy. 2 ml of D10 and R10 culture media were mixed with 10 μl of beads, and the bioluminescence values were measured from isolated beads that were resuspended in 100 μl of PBST (D10 with beads and R10 with beads) as described above. The bioluminescence values from 100 μl of the two cultured media (D10 and R10) were measured simultaneously. (F) and (G) Enhancement of the purification strategy on the linear correlation of secNluc and emitted bioluminescence. The culture medium containing the secreted secNluc-FLAG protein was diluted from 0- to 1024-fold in D10 or R10 media, and 100 μl of the diluted solutions and 100 μl of the resuspended solutions that were obtained from the corresponding diluted solutions using the purification strategy were used to measure the emitted bioluminescence (F). 2 × 10^1^ to 2 × 10^5^ transfected cells per well were inoculated into 24-well plates for 24 h, and 100 μl of the cultured media and 100 μl of the resuspended solutions obtained from the corresponding cultured media using the purification strategy were used to measure the emitted bioluminescence (G). (H) The elimination of interference from glycyrrhizic acid using the purification strategy. Glycyrrhizic acid at concentrations from 0 to 100 μg/ml was added to D10 media containing the secNluc-FLAG protein. 100 μl of media and 100 μl of the resuspended solutions obtained from the corresponding media using the purification strategy were used to measure the emitted bioluminescence. (I) Effects of secNluc with or without FLAG-tag in different pH values on secNluc-catalyzed bioluminescence reactions. The assay was performed as described in the Materials and Methods. (J) Effects of secNluc with or without FLAG-tag in different drug monomer on secNluc-catalyzed bioluminescence reactions. Because of the extensive dilution multiple, Logarithmic calculation was employed in order to decrease the drift degree of data. In (B) to (J), the data shown are the means ± standard errors of three independent experiments.

Since interfering factors in the medium could affect secNluc-catalyzed bioluminescence reactions, we tried to develop a method to resolve these problems. Magnetic beads conjugated with an antibody against a specific protein tag could recognize and catch secNluc that was fused with the tag and thus separate the enzyme from the surrounding medium through a simple step consisting of magnet attraction and supernatant removal. This process could get rid of interfering factors in the medium and transfer secNluc into an optimal buffer, thus improving the application of the secNluc reporter system. Herein, we fused a FLAG tag to the C-terminus of secNluc using genetic construction (Fig 3A) and used anti-FLAG magnetic beads to carry out the purification strategy.

We first examined whether the beads themselves could produce luminescence. The result presented in Fig 3 showed no differences in luminescence signals from samples in both the presence and absence of beads, indicating that the beads had no background signal. The result presented in Fig 3C confirmed that for 100 μl of media, 2.5 μl of beads could recover almost all of the secNluc-FLAG proteins. For 2 and 5 ml of media, 10 μl of beads could recover approximately 90% and 80% of secNluc-FLAG proteins, respectively, and 2.5 μl of beads could recover approximately 65% and 50% of secNluc-FLAG proteins, respectively (Fig 3D). This result indicated that secNluc-FLAG proteins could be concentrated dozens of times from 2 and 5 ml of solution if they were put in 100 μl of solution. As proof, RLU values from 2 ml of DMEM and RPMI1640 using 10 μl of beads were approximately 18.4-fold and 17.5-fold higher than those from 100 μl of those media in our test, respectively (Fig 3E). Notably, the recover abilities among the four media were not obviously different (Fig 3C). Subsequent examination showed that similar to the result above (Fig 1C), when the medium containing secNluc-FLAG was diluted with a certain fold D10 or R10, which represented a certain low concentration of secNluc-FLAG in medium, the RLU values tended to be constant due to the background FBS signal (Fig 3F). When using the purification method, however, the RLU values exhibited a significant linear correlation with the dilution fold till the value approached zero (the lower detection limit of the apparatus) (Fig 3F). Another test revealed a similar result as seen in Fig 3G. The RLU values in the group with beads purification had good linear correlation with the inoculated cell numbers throughout the entire test. In the group without bead purification, however, the RLU values tended to be constant due to the background FBS signal when the inoculated cell numbers were less than 2 × 10^3^ cells per well in this test. In addition, the result presented in Fig 3H showed that the bead purification method could help restore the luciferase activity of secNluc-FLAG that was inhibited by glycyrrhizic acid, a residual reagent mimic (Fig 1D).

There was a possibility that the fused FLAG-tag in this strategy may had the stabilizing effect on the enzymatic activity of secNluc. We thus tested the influence of FLAG-tag by using secNluc and secNluc-FLAG in this study. The results showed that RLU had no significant difference even though FLAG-tag existed or not at each pH value (Fig 3I), demonstrating that secNluc-catalyzed bioluminescence reactions was not impacted by FLAG-tag under different pH value. Furthermore, secNluc-FLAG did not show any stabilizing effect on secNluc-catalyzed bioluminescence reactions in the presence of drug monomers (Fig 3J).

### The purification strategy optimizes TNF-α-stimulated NF-κB activation assay—A case study

**Fig 4:**
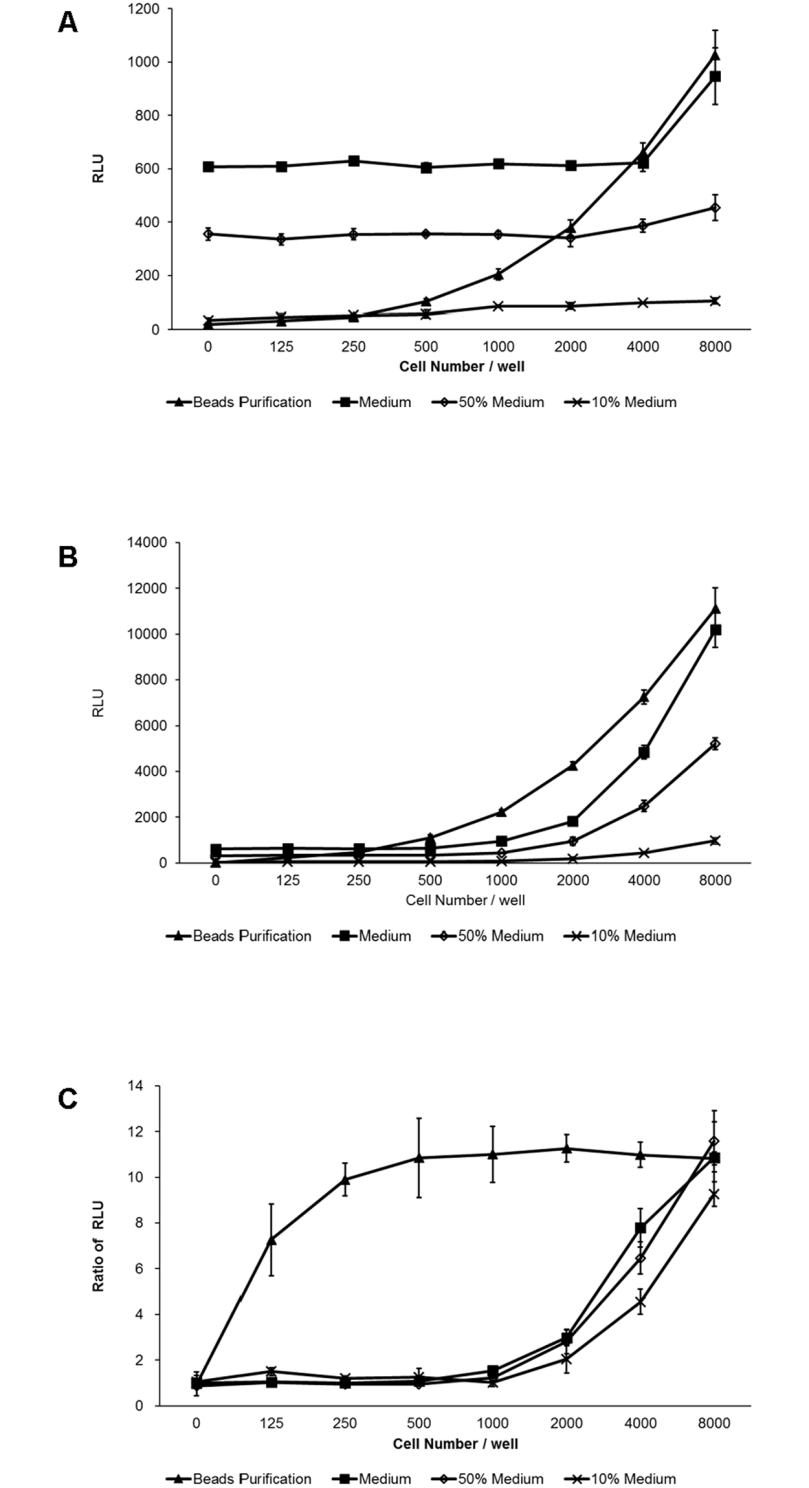
TNF-α-stimulated NF-κB activation assay. (A) and (B) Measurement of bioluminescence from cells without or with TNF-αstimulation. Monoclonal HeLa cells stably transfected with the NF-κB-secNluc-FLAG gene were inoculated at different densities (0 to 8000 cells per well) in 24-well plates for 12 h. For TNF-α stimulation, 50 ng/ml TNF-α was added for 12 h to induce the activation of the NF-κB signaling pathway (B). 100 μl of each cultured medium was subjected to magnetic bead purification, and the beads were resuspended in 100 μl of PBST. 100 μl of resuspended solution (Beads Purification), 100 μl of cultured medium (Medium), 50 and 10 μl of culture media that were supplemented with PBST to 100 μl (50% Medium and 10% Medium) were used to measure the emitted bioluminescence. The experiments were repeated three times with comparable results. (C) The ratios of RLU in groups with TNF-α stimulation to those in groups without TNF-α stimulation. The data shown are the means ± standard errors of three independent experiments.

We used the TNF-α-stimulated NF-κB activation assay as a case study to examine the improved effects resulting from our purification strategy. The RLU values in the Medium group were maintained at approximately 600 units when a small number of inoculated cells were used (less than 4000 cells per well without TNF-α stimulation and less than 500 cells per well with TNF-α stimulation) (Fig 4A and 4B), which led to a low increase in signals responding to TNF-α stimulation when inoculated cells were less than 4000 per well in this test (Fig 4C). In the Beads Purification group, however, the increase in signals responding to TNF-α stimulation was rather high, even when the inoculated cells were the fewest 125 per well in this test (Fig 4C). The fold of increase tended to be constant when inoculated cells were more than 250 per well in this test (Fig 4C). Moreover, although diluting the medium with PBST (50% Medium group and 10% Medium group) could lower the background signal (Fig 4A and 4B), the increases in signals responding to TNF-α stimulation were not improved compared to that of the Medium group when inoculated cells were less than 8000 per well in this test (Fig 4C).

## Discussion

Secreted luciferases have significant advantages over intracellular luciferases as reporters in non-disruptive reporter gene assays, real-time monitoring and high-throughput drug screening [19–24]. The benefits of secreted luciferases also include their applications in *ex vivo* monitoring of *in vivo* biological processes and in providing dual luciferase assays together with intracellular luciferases[3,28,29]. Nevertheless, secreted luciferases have several disadvantages[25], as they are inevitably mixed with components of conditioned medium surrounding test cells or biological samples, such as blood or urine[26]. These mixtures may affect the results of luciferase-catalyzed bioluminescence reactions, especially in experiments requiring superior sensitivity, low background noise and high reproducibility. Therefore, eliminating interfering components and transferring luciferase-catalyzed bioluminescence reactions into an optimized buffer should lead to more qualified results.

As a secreted luciferase, secNluc was newly developed for bioluminescence application. Compared with other secreted luciferases such as *Gaussia*, *Metridia* and *Cypridina* luciferases, Nano-luciferase possesses the advantages of high structural stability, long half-life, and glow-type kinetics together with high light emission intensity, and thus would become one of the most valuable tools for bioluminescence assays[12,13]. However, the application of secNluc can similarly be affected by the environment. In this study, we first tested the effects of three factors in conditioned medium, pH, serum and residual reagents, on the secNluc-catalyzed bioluminescence reactions using coelenterazine as the substrate. The pH of the conditioned medium is known to often be influenced by lactic acid, the by-product of cellular metabolism[30]. The pH of a normal mammalian cell culture medium may shift from an optimal pH of 7.4 to acidic pH of 6.5 or below when cells are cultured at high density or are overgrown. Our results showed that lower pH values significantly decreased the intensity of secNluc-catalyzed bioluminescence reactions (Fig 1B). Serum is commonly used to support the growth of cells in culture. In our study, the existence of fetal bovine serum resulted in a degree of background signal, which significantly affected bioluminescence measurements in experiments with low cell numbers (Fig 1C). This phenomenon was consistent with the previously reported observations that the presence of serum from different species in the assay medium resulted in significant increases in luminescent signal [25,31]. Several medicines were employed in this study as reagents to evaluate the probable interferences of residual reagents on secNluc-catalyzed bioluminescence reactions. Some, although not all, of these reagents greatly decreased the luminescence signal (Fig 1D). Replacing culture medium with fresh medium to eliminate the reagent can of course minimize the interference. However, this process is not only laborious and time-consuming but may also weaken the experimental effects induced by reagents due to having to wait hours for the accumulation of the newly secreted luciferase without the administration of reagents. Overall, these results indicated that the factors pH, serum and residual reagents that exist in the environment in which secNluc remains after secretion could interfere bioluminescence reactions and/or result in background signal, thus affecting the precision and reliability of the secNluc reporter system.

To resolve these issues, in this study, we raised a simple affinity purification strategy in which we fused a FLAG-tag onto the C-terminus of secNluc using plasmid construction to produce a recombinant secNluc-FLAG protein (Table 1). When applying this reporter system, we used anti-FLAG magnetic beads and a magnet to capture the recombinant proteins from cultured medium and transfer them directly into an optimal buffer before measuring light emission as usual. Herein, PBS (pH 7.4) supplemented with 0.03% (v/v) Triton X-100 was used as the reaction buffer, and stable light emission was more achievable in this buffer than in PBS or in media with or without FBS (Fig 2). Nevertheless, other optimal buffers can also be used with our strategy if they provide a stable pH and contribute to the production of low-background and stable light emission. Our results showed that through the purification process, in addition to the optimal buffer resulted in accurate and reproducible measurements, the bioluminescence detection limit and the number of cells required for detection were greatly lowered due to dramatically decreased background signal (Fig 3F and 3G), and meanwhile interference from residual reagents was eliminated (Fig 3H). Because the magnetic beads themselves had no light output (Fig 3B) and had no obvious harm to the bioluminescence reactions based on the approximate 100% recovery rate (Fig 3C), they can be reserved in the reaction system. Thus, the simplest purification process could only comprise one time of mixture of medium and beads, and one or two times of collection of magnetic beads by magnet, lasting only a few minutes.

Furimazine was manufactured by Promega as a modified substrate for Nano-luciferases, including secNluc [12]. It gives a higher initial bioluminescence signal and a much slower signal decay compared to coelenterazine. However, regardless of its far more expensive cost, enzyme-catalyzed bioluminescence reactions using this substrate are also affected by lower pH conditions[12], and meanwhile background signal caused by serum and interference brought from residual reagents cannot also be ignored. Therefore, the purification strategy presented herein may also be suitable for secNluc reporter systems using furimazine.

Another advantage of this strategy is the enablement of enriching secNluc from conditioned medium or biological samples, such as blood or urine. Our results showed that most of secNluc in volumes of 2.5 ml or 5 ml could be captured and enriched in a volume of 100 μl (Fig 3D), which can be loaded into a well of 96-well plates for bioluminescence measurement. This enrichment would be necessary when secNluc concentrations are lower than the limit of detection, such as when enzymes are secreted from a small number of cells, when enzyme expression is driven by a weak promotor, or when secreted enzymes are diluted in a large volume of biological sample, such as blood or urine.

The transcription activation assay is one of the most common applications of secreted luciferases. Herein, we used the TNF-stimulated NF-κB activation assay as a case study to examine the improved effects of our strategy. The results indicated that when the measured signals were weak, the increased fold of NF-κB activation induced by TNF-α stimulation using the bead purification strategy was far higher than those observed without the strategy (Fig 4C). This improvement thus most likely represents the effect of TNF stimulation and may contribute to some investigations, such as the discovery of rare natural drugs.

The luciferase reporter system is nowadays accepted as a favored and valuable tool for high-throughput drug screening [9]. Reliability in high-throughput screening requires the maximum parallelization of the detection process. In the application of the luciferase reporter system, however, the parallelism may be easily affected by the rapid decay of the bioluminescence signal, the interfering components existing in samples, such as serum or residual reagents, and so on. The strategy developed in this study presents an optimized application of secreted luciferase-catalyzed bioluminescence analysis by eliminating the interference of reagents and residual substances and providing an optimized and uniform environment for bioluminescence reactions, and thus may benefit the high-throughput drug screening using the luciferase reporter system.

In summary, we have developed a simple affinity purification strategy by which various optimizations of the application of the secNluc reporter system are simultaneously permitted. This strategy is not only suitable for the secNluc reporter system but also for reporter systems involving other secreted luciferases because the environmental interferences and enhancements from reaction buffer optimization and enzyme enrichment are basically common among all secreted luciferase reporter systems. We hope this strategy will contribute to biomedical studies utilizing secreted luciferases, especially those requiring superior sensitivity, low background noise and high reproducibility.

## References

[1] Greer LF 3rd, Szalay AA (2002) Imaging of light emission from the expression of luciferases in living cells and organisms: a review. Luminescence 17: 43–74. doi: 10.1002/bio.676 11816060 10.1002/bio.676

[2] Kaskova ZM, Tsarkova AS, Yampolsky IV (2016) 1001 lights: luciferins, luciferases, their mechanisms of action and applications in chemical analysis, biology and medicine. Chem Soc Rev 45: 6048–6077. doi: 10.1039/c6cs00296j 27711774 10.1039/c6cs00296j

[3] Wurdinger T, Badr C, Pike L, de Kleine R, Weissleder R, Breakefield XO, et al (2008) A secreted luciferase for ex vivo monitoring of in vivo processes. Nat Methods 5: 171–173. doi: 10.1038/nmeth.1177 18204457 10.1038/nmeth.1177 PMC2699561

[4] Badr CE, Wurdinger T, Tannous BA (2011) Functional drug screening assay reveals potential glioma therapeutics. Assay Drug Dev Technol 9: 281–289. doi: 10.1089/adt.2010.0324 21184646 10.1089/adt.2010.0324 PMC3102258

[5] Pichler A, Prior JL, Luker GD, Piwnica-Worms D (2008) Generation of a highly inducible Gal4—>Fluc universal reporter mouse for in vivo bioluminescence imaging. Proc Natl Acad Sci U S A 105: 15932–15937. doi: 10.1073/pnas.0801075105 18843112 10.1073/pnas.0801075105 PMC2572923

[6] Niers JM, Kerami M, Pike L, Lewandrowski G, Tannous BA (2011) Multimodal in vivo imaging and blood monitoring of intrinsic and extrinsic apoptosis. Mol Ther 19: 1090–1096. doi: 10.1038/mt.2011.17 21343914 10.1038/mt.2011.17 PMC3129810

[7] Sacco A, Doyonnas R, Kraft P, Vitorovic S, Blau HM (2008) Self-renewal and expansion of single transplanted muscle stem cells. Nature 456: 502–506. doi: 10.1038/nature07384 18806774 10.1038/nature07384 PMC2919355

[8] Bhang HE, Gabrielson KL, Laterra J, Fisher PB, Pomper MG (2011) Tumor-specific imaging through progression elevated gene-3 promoter-driven gene expression. Nat Med 17: 123–129. doi: 10.1038/nm.2269 21151140 10.1038/nm.2269 PMC3057477

[9] Fan F, Wood KV (2007) Bioluminescent assays for high-throughput screening. Assay Drug Dev Technol 5: 127–136. doi: 10.1089/adt.2006.053 17355205 10.1089/adt.2006.053

[10] Massoud TF, Paulmurugan R, De A, Ray P, Gambhir SS (2007) Reporter gene imaging of protein-protein interactions in living subjects. Curr Opin Biotechnol 18: 31–37. doi: 10.1016/j.copbio.2007.01.007 17254764 10.1016/j.copbio.2007.01.007 PMC4141564

[11] Degeling MH, Bovenberg MS, Lewandrowski GK, de Gooijer MC, Vleggeert-Lankamp CL, Tannous M, et al (2013) Directed molecular evolution reveals Gaussia luciferase variants with enhanced light output stability. Anal Chem 85: 3006–3012. doi: 10.1021/ac4003134 23425213 10.1021/ac4003134 PMC3617556

[12] Hall MP, Unch J, Binkowski BF, Valley MP, Butler BL, Wood MG, et al (2012) Engineered luciferase reporter from a deep sea shrimp utilizing a novel imidazopyrazinone substrate. ACS Chem Biol 7: 1848–1857. doi: 10.1021/cb3002478 22894855 10.1021/cb3002478 PMC3501149

[13] England CG, Ehlerding EB, Cai W (2016) NanoLuc: A Small Luciferase Is Brightening Up the Field of Bioluminescence. Bioconjug Chem 27: 1175–1187. doi: 10.1021/acs.bioconjchem.6b00112 27045664 10.1021/acs.bioconjchem.6b00112 PMC4871753

[14] Germain-Genevois C, Garandeau O, Couillaud F (2016) Detection of Brain Tumors and Systemic Metastases Using NanoLuc and Fluc for Dual Reporter Imaging. Mol Imaging Biol 18: 62–69. doi: 10.1007/s11307-015-0864-2 26002233 10.1007/s11307-015-0864-2

[15] Machleidt T, Woodroofe CC, Schwinn MK, Mendez J, Robers MB, Zimmerman K, et al (2015) NanoBRET—A Novel BRET Platform for the Analysis of Protein-Protein Interactions. ACS Chem Biol 10: 1797–1804. doi: 10.1021/acschembio.5b00143 26006698 10.1021/acschembio.5b00143

[16] Schaub FX, Reza MS, Flaveny CA, Li W, Musicant AM, Hoxha S, et al (2015) Fluorophore-NanoLuc BRET Reporters Enable Sensitive In Vivo Optical Imaging and Flow Cytometry for Monitoring Tumorigenesis. Cancer Res 75: 5023–5033. doi: 10.1158/0008-5472.CAN-14-3538 26424696 10.1158/0008-5472.CAN-14-3538 PMC4668208

[17] Song G, Wu QP, Xu T, Liu YL, Xu ZG, Zhang SF, et al (2015) Quick preparation of nanoluciferase-based tracers for novel bioluminescent receptor-binding assays of protein hormones: Using erythropoietin as a model. J Photochem Photobiol B 153: 311–316. doi: 10.1016/j.jphotobiol.2015.10.014 26506452 10.1016/j.jphotobiol.2015.10.014

[18] Stacer AC, Nyati S, Moudgil P, Iyengar R, Luker KE, Rehemtulla A, et al (2013) NanoLuc reporter for dual luciferase imaging in living animals. Mol Imaging 12: 1–13. PMC4144862 24371848

[19] Chung E, Yamashita H, Au P, Tannous BA, Fukumura D, Jain RK (2009) Secreted Gaussia luciferase as a biomarker for monitoring tumor progression and treatment response of systemic metastases. PLoS One 4: e8316 doi: 10.1371/journal.pone.0008316 20016816 10.1371/journal.pone.0008316 PMC2789383

[20] Liu M, Blinn C, McLeod SM, Wiseman JW, Newman JV, Fisher SL, et al (2014) Secreted Gaussia princeps luciferase as a reporter of Escherichia coli replication in a mouse tissue cage model of infection. PLoS One 9: e90382 doi: 10.1371/journal.pone.0090382 24595353 10.1371/journal.pone.0090382 PMC3942414

[21] Yamashita H, Nguyen DT, Chung E (2014) Blood-based assay with secreted Gaussia luciferase to monitor tumor metastasis. Methods Mol Biol 1098: 145–151. doi: 10.1007/978-1-62703-718-1_12 24166375 10.1007/978-1-62703-718-1_12

[22] Michelini E, Cevenini L, Calabretta MM, Calabria D, Roda A (2014) Exploiting in vitro and in vivo bioluminescence for the implementation of the three Rs principle (replacement, reduction, and refinement) in drug discovery. Anal Bioanal Chem 406: 5531–5539. doi: 10.1007/s00216-014-7925-2 24908412 10.1007/s00216-014-7925-2

[23] Charles JP, Fuchs J, Hefter M, Vischedyk JB, Kleint M, Vogiatzi F, et al (2014) Monitoring the dynamics of clonal tumour evolution in vivo using secreted luciferases. Nat Commun 5: 3981 doi: 10.1038/ncomms4981 24889111 10.1038/ncomms4981 PMC4059931

[24] Alessandrini F, Ceresa D, Appolloni I, Marubbi D, Malatesta P (2016) Noninvasive Monitoring of Glioma Growth in the Mouse. J Cancer 7: 1791–1797. doi: 10.7150/jca.15564 27698917 10.7150/jca.15564 PMC5039361

[25] Badr CE (2014) Bioluminescence imaging: basics and practical limitations. Methods Mol Biol 1098: 1–18. doi: 10.1007/978-1-62703-718-1_1 24166364 10.1007/978-1-62703-718-1_1

[26] Tannous BA, Teng J (2011) Secreted blood reporters: insights and applications. Biotechnol Adv 29: 997–1003. doi: 10.1016/j.biotechadv.2011.08.021 21920429 10.1016/j.biotechadv.2011.08.021 PMC3189544

[27] Geisse S, Jordan M, Wurm FM (2005) Large-scale transient expression of therapeutic proteins in mammalian cells. Methods Mol Biol 308: 87–98. doi: 10.1385/1-59259-922-2:087 16082028 10.1385/1-59259-922-2:087

[28] Morita N, Haga S, Ohmiya Y, Ozaki M (2016) Long-term ex vivo and in vivo monitoring of tumor progression by using dual luciferases. Anal Biochem 497: 24–26. doi: 10.1016/j.ab.2015.12.007 26717897 10.1016/j.ab.2015.12.007

[29] El-Amouri SS, Cao P, Miao C, Pan D (2013) Secreted luciferase for in vivo evaluation of systemic protein delivery in mice. Mol Biotechnol 53: 63–73. doi: 10.1007/s12033-012-9519-6 22407720 10.1007/s12033-012-9519-6 PMC4040271

[30] Naciri M, Kuystermans D, Al-Rubeai M (2008) Monitoring pH and dissolved oxygen in mammalian cell culture using optical sensors. Cytotechnology 57: 245–250. doi: 10.1007/s10616-008-9160-1 19003181 10.1007/s10616-008-9160-1 PMC2570003

[31] Zhao H, Doyle TC, Wong RJ, Cao Y, Stevenson DK, Piwnica-Worms D, et al (2004) Characterization of coelenterazine analogs for measurements of Renilla luciferase activity in live cells and living animals. Mol Imaging 3: 43–54. doi: 10.1162/153535004773861714 15142411 10.1162/15353500200403181

